# Molecular interactions, structural effects, and binding affinities between silver ions (Ag^+^) and amyloid beta (Aβ) peptides

**DOI:** 10.1038/s41598-024-59826-6

**Published:** 2025-02-13

**Authors:** Amanda L. Lakela, Elina Berntsson, Faraz Vosough, Jüri Jarvet, Suman Paul, Andreas Barth, Astrid Gräslund, Per M. Roos, Sebastian K. T. S. Wärmländer

**Affiliations:** 1 Department of Biochemistry and Biophysics, Arrhenius Laboratories, Stockholm University, 10691 Stockholm, Sweden; 2 CellPept Sweden AB, Kvarngatan 10B, 11847 Stockholm, Sweden; 3 Department of Chemistry and Biotechnology, Tallinn University of Technology, 19086 Tallinn, Estonia; 4 The National Institute of Chemical Physics and Biophysics, Tallinn, Estonia; 5 Institute of Environmental Medicine, Karolinska Institutet, 17177 Stockholm, Sweden; 6 University Healthcare Unit of Capio St. Göran Hospital, 11281 Stockholm, Sweden; 7 Chemistry Section, Arrhenius Laboratories, Stockholm University, 10691 Stockholm, Sweden

**Keywords:** Alzheimer’s disease, Amyloid aggregation, Metal-protein binding, Neurodegeneration, Metal toxicity, Spectroscopy, Biophysical chemistry, Metals, Neurochemistry, Peptides, Proteins

## Abstract

Because silver is toxic to microbes, but not considered toxic to humans, the metal has been used as an antimicrobial agent since ancient times. Today, silver nanoparticles and colloidal silver are used for antibacterial purposes, and silver-peptide and similar complexes are being developed as therapeutic agents. Yet, the health effects of silver exposure are not fully understood, nor are the molecular details of silver-protein interactions. In Alzheimer’s disease, the most common form of dementia worldwide, amyloid-β (Aβ) peptides aggregate to form soluble oligomers that are neurotoxic. Here, we report that monovalent silver ions (Ag^+^) bind wildtype Aβ_40_ peptides with a binding affinity of 25 ± 12 µM in MES buffer at 20 °C. Similar binding affinities are observed for wt Aβ_40_ peptides bound to SDS micelles, for an Aβ_40_(H6A) mutant, and for a truncated Aβ(4–40) variant containing an ATCUN (Amino Terminal Cu and Ni) motif. Weaker Ag^+^ binding is observed for the wt Aβ_40_ peptide at acidic pH, and for an Aβ_40_ mutant without histidines. These results are compatible with Ag^+^ ions binding to the N-terminal segment of Aβ peptides with linear bis-his coordination. Because the Ag^+^ ions do not induce any changes in the size or structure of Aβ_42_ oligomers, we suggest that Ag^+^ ions have a minor influence on Aβ toxicity.

## Introduction

Alzheimer’s disease (AD) is one of the most common neurodegenerative diseases^1,2^. In addition to old age, the main AD risk factors include genetic factors, such as Down’s syndrome and unfavorable alleles of the ApoE gene and, environmental factors such as smoking, and other medical conditions such as diabetes and traumatic brain injury^1–4^. Many drug candidates to mitigate AD symptoms have been proposed, however with moderate success^5–8^. Identifying new molecular targets to treat and diagnose the disease therefore remains imperative^9,10^.

At the molecular level, the main event underlying AD progression is aggregation of amyloid-β (Aβ) peptides and tau proteins, first into toxic soluble oligomers^11–13^, and then into insoluble tangles and/or amyloid fibrils^14^. These aggregates eventually deposit as plaques in the brains of AD patients^2,15^. Aβ peptides of different lengths are created from the amyloid-β precursor protein, APP, via cleavage by β- and γ-secretase enzymes^16^. The two most common Aβ variants in humans are Aβ(1–40) and Aβ(1–42), here abbreviated Aβ_40_ and Aβ_42_, respectively^17^. Monomers of both variants are intrinsically disordered in aqueous solution, but can adopt ordered secondary structures such as β-sheet^18^ and α-helix^19^ in non-aqueous environments^20,21^. The β-sheet structure is particularly important, as Aβ peptides in β-sheet hairpin conformations are the building blocks of the aggregated fibrils^18^.

The expression, accumulation, and aggregation of different Aβ peptides can be influenced by various interacting factors^3,22^ including cationic molecules^23^, metal ions^24–26^, and other proteins and peptides^8,27–29^, some of which are amyloid-forming in themselves^30–34^.

Different metal ions have been found deposited in the amyloid plaques of AD brains, suggesting that metal ions could be involved in AD pathogenesis and/or progression^35–37^. Both endogenous and exogenous metal ions are therefore of special interest in AD pathology. Known molecular mechanisms for metal toxicity include molecular and ionic mimicry^38^, but other mechanisms are also possible, such as modulation of protein expression, modification, misfolding, or aggregation^3,39–44^.

Silver (Ag) is a common metal in human society, but it has no known biological function in humans or other mammals^45^. The metal is not considered very toxic to humans^44^, but is highly toxic to bacteria^46,47^. The toxic mechanisms are unclear^48^. Silver ions can readily bind to proteins and peptides^49–52^, often at similar binding sites as Cu^+^ ions^53–58^. It has previously been shown that Ag^+^ binding to Aβ_40_ peptides retards their aggregation and possibly changes their secondary structures^26,59,60^. However, many aspects of the molecular interactions between Ag^+^ ions and Aβ peptides are still unknown, especially for other peptide variants than Aβ_40_.

Here, we use biophysical spectroscopic and imaging techniques to investigate the binding interactions between Ag^+^ ions and different variants of Aβ peptides, with a focus on characterizing binding affinities and structural effects.

## Biochemical and toxicological aspects of silver

Because Ag is toxic to microbes, but not considered toxic to humans, metallic Ag has been used as an antimicrobial agent since ancient times^47,48,61^. During the last decades, various types of Ag nanoparticles have been developed as a new generation of antimicrobials^48,61,62^. Such particles are now increasingly used in e.g. skin creams, impregnated wound care dressing, and eye drops^45^. Interestingly, tyrosine-coated Ag nanoparticles have been found to inhibit amyloid aggregation of insulin, suggesting that such modified nanoparticles might be used to combat amyloid diseases^63^. In addition to nanoparticles, Ag-peptide complexes have been tried as therapeutic agents^51,64^.

Silver has furthermore found its way into homeopathy, both in a product known as Argentum Nitricum, and as nanoscale Ag particles suspended in liquid (i.e., colloidal silver), which have been suggested to possess anti-inflammatory, antibacterial, and antiviral properties^65^. Significant interest has also been directed towards potential anti-cancer activities of Ag nanoparticles^66^. The scientific evidence supporting all these claims is however relatively weak^67^.

Several routes of exposure exist for Ag. Dermal exposure occurs mainly via jewellery and via antimicrobial impregnation of clothing. Enteric Ag exposure occurs through water filters, dental restoration materials, and confectionary sugar pearls—Ag is a legal food additive within EU regulations. Coal combustion contributes to the total Ag exposure via the respiratory route, as does cloud seeding with crystalline Ag iodide particles for rain control^68,69^.

Silver accumulates in tissues and extended Ag exposure can produce a pseudocyanotic state with changes in skin pigmentation and blueish discoloration of gums and viscera, a condition known as argyria^70^. Silver penetrates both the blood–brain barrier and the placental barrier^71^. The metal is classified as a sequestered choroid plexus toxicant in relation to its ability to penetrate the barriers, and in that respect behaves similar to iron, zinc, and gold^72^. Thus, Ag is stored in the choroid plexus structures without destroying the blood-CSF barrier. Long-time Ag exposure in one argyria case showed at autopsy Ag accumulation in the choroid plexus, but no accumulation in the brain^73^. Yet, Ag nanoparticles have been reported to penetrate from the bloodstream to the brain, leading to accumulation in the brain over time^74^. Silver particles have also been shown to accumulate in the liver^75^. Elimination of Ag is mainly biliary, with a relatively short half-life in the human body of one day up to a month^45^.

Acute human Ag toxicity is low, with toxic effects shown mainly from the cardiovascular system, haematopoiesis, and the hepatic clearance pathways. Silver neurotoxicity is scarcely reported, although prolonged intraperitoneal Ag exposure has been found to produce Ag deposition in rat brain and spinal cord^76^. Formation of silver sulphide (Ag_2_S) deposits have been found next to cutaneous nerves, but without any obvious toxicity towards the peripheral nervous system^77^. For Ag particles the toxicity depends on the size, where smaller particles elicit a more profound tissue response. The extent to which metallic Ag particles dissolve into free Ag ions increases with smaller particle sizes, and below nanoscale the distinction between particles and ions blurs^78^.

Overall, Ag toxicity to the brain seems to be low with efficient biliary excretion systems, short half-life in the body, and protection from Ag by an unharmed blood-CSF barrier^45^. But silver nanoparticles penetrate easily into the brain, and the extent of their neurotoxicity is currently under intense debate^48,62,79–81^.

## Materials and methods

### Materials

Sodium dodecyl sulfate (SDS) detergent was bought from ICN Biomedicals Inc (USA). AgNO_3_ salt and 2-(N-Morpholino)ethanesulfonic acid hydrate (MES) buffer were purchased from Sigma (Sigma/Merck KGaA, Darmstadt, Germany).

Synthetic lyophilized wildtype (wt) Aβ(1–42) peptide, abbreviated Aβ_42_, with the primary sequence DAEFR_5_HDSGY_10_EVHHQ_15_KLVFF_20_AEDVG_25_SNKGA_30_IIGLM_35_VGGVV_40_IA, was purchased from JPT Peptide Technologies GmbH (Germany). In addition, a number of recombinantly produced Aβ variants in lyophilized form were purchased from AlexoTech AB (Umeå, Sweden): wild-type Aβ_40_ peptides, Aβ_40_(H6A) mutant, Aβ_40_(H6A, H13A, H14A) mutant, truncated Aβ(4–40) peptide. and uniformly^15^N-labeled Aβ_40_ wt peptide. The Aβ_40_(H6A, H13A, H14A) mutant will in this study be referred to as the Aβ_40_(NoHis) variant. All Aβ_40_ variants were stored at − 80 °C until used. Immediately before each measurement the powder of the Aβ_40_ peptide variants was dissolved to monomeric form in 10 mM NaOH at pH 12, 1 mg/mL peptide concentration. The solution was then sonicated for three minutes in an ice-bath to dissolve any pre-formed aggregates. All peptide solutions were further diluted in either MES or sodium phosphate buffer, to final Aβ concentrations of 10 µM for the AFM, CD, and fluorescence experiments, and to 92.4 µM for the NMR experiments (see below). The sample preparation steps were performed on ice, and the peptide concentrations were first estimated from the dry weight of the powder, and then more accurately determined by measurements with a NanoDrop Microvolume Spectrophotometer (Thermo Fisher Scientific Inc).

### Preparation of Aβ_42_ oligomers

Earlier work has shown that incubation of Aβ_42_ peptides with low concentrations (≤ 7 mM) of SDS, i.e. below the SDS critical micelle concentration which in pure water at 25 °C is 8.2 mM^82^, can produce stable and homogeneous Aβ_42_ oligomers of defined sizes and conformations^83–85^. Here, we prepared one type of such oligomers according to our previously reported protocol^85^.

First, synthetic Aβ_42_ peptides were purified into monomers via size exclusion chromatography (SEC). Initially, 1 mg of lyophilized Aβ_42_ powder was dissolved in 250 µL of DMSO. Then, a Sephadex G-250 HiTrap desalting column (GE Healthcare, Uppsala) was washed with a solution of NaOH (5 mM; pH = 12.3), and equilibrated with 10–15 mL NaOD solution (5 mM; pD = 12.7)^86^. The solution of Aβ_42_ peptide in DMSO (0.25 ml, 4 mg/mL) was applied to the column, followed by injection of NaOD (1.25 mL; 5 mM). Ten peptide fractions in 5 mM NaOD (0.1 mL volume each) were collected at a 1 mg/mL flow rate and stored in 1.5 mL Eppendorf tubes on ice. A NanoDrop instrument (Eppendorf, Germany) was used to record the absorbance for each fraction at 280 nm, allowing peptide concentrations to be determined using the molar extinction coefficient of 1280 M^-1^ cm^-1^ for Tyr10^87^. The monomeric peptide samples were flash-frozen in liquid N_2_, covered with Argon gas on top in the Eppendorf tubes, and stored at – 80 °C until used.

The Aβ_42_ monomers were then used to prepare SDS-stabilized Aβ_42_ oligomers of approximately tetrameric size, using a previously published protocol^83^ with the following modifications: the oligomers were prepared in D_2_O, at four-fold lower peptide concentration, and without the original dilution step^85^. The reaction mixture (100–120 μM Aβ_42_ in D_2_O-based PBS, containing 0.2% (6.9 mM) SDS) was incubated at 37 °C for 24 h together with either 0, 10, 100, or 500 µM AgNO_3_, respectively, and then flash-frozen in liquid N_2_ and stored at –20 °C for later analysis.

### AFM imaging

Samples for AFM imaging were produced by incubating 10 µM Aβ_40_ peptides in 5 mM MES buffer, pH 7.3, for 72 h at 37 °C with continuous shaking at 350 rpm together with different amounts of AgNO_3_, i.e. 0 µM; 2.5 µM; 5 µM; and 30 µM (corresponding to Ag^+^:Aβ ratios of 0:1; 1:4; 1:2; and 3:1, respectively). The incubated samples were placed as 1 µL droplets on fresh silicon wafers (Siegert Wafer GmbH, Germany). After 2 min, 10 µL of Milli-Q water was added to the droplets, and then a lint-free wipe was used to remove all excess fluid. Next, the wafers were left to air-dry in a container, covered to protect from dust, and later the same day AFM images were recorded. A neaSNOM AFM unit (Neaspec GmbH, Germany) was used, running in tapping mode (Ω: 280 kHz, tapping amplitude 50–55 nm) and equipped with Pt/Ir-coated monolithic ARROW-NCPt Si tips (NanoAndMore GmbH, Germany) where the tip radius was less than 10 nm. Both topographic and mechanical phase images were recorded for 2.5 × 2.5 µm scanning areas (pixel size 200 × 200) with a scan speed of 2.5 ms/pixel. The images were minimally processed to perform basic plane levelling using the Gwyddion software^88^.

### NMR spectroscopy

NMR spectrometry measurements were performed on a 700 MHz Advance instrument (Bruker GmbH., Germany) equipped with a cryoprobe for improved sensitivity. Two-dimensional ^1^H,^15^N-HSQC spectra were recorded at 5 °C for 92.4 µM ^15^N-labeled Aβ_40_ peptide in 20 mM MES buffer (90/10 H_2_O/D_2_O), at either pH 5.1 or pH 7.4. For both samples, silver nitrate was added in steps of 37 µM (0.4:1 Ag^+^:Aβ ratio), 92.4 µM (1:1 ratio), and 277.2 µM (3:1 ratio). The sample volume was 500 µL, and the NMR spectra were referenced to TSP (trimethylsilylpropanoic acid) and then processed with the Topspin v. 3.6.2 software (Bruker GmbH., Germany). The assignment of the ^1^H,^15^N-HSQC spectrum for Aβ_40_ peptides in aqueous solution is known from earlier studies, both at neutral pH^89–91^ and at acidic pH^92^.

### CD spectroscopy

Circular dichroism (CD) spectroscopy was performed with a Chirascan CD instrument (Applied Photophysics Ltd., U.K.) using a quartz cuvette with 2 mm pathlength containing 600 µL of 10 µM Aβ_40_ peptide in 20 mM phosphate buffer, pH 7.3. CD spectra were recorded at 20 °C between 190 and 260 nm, with 0.5 nm step size. One sample contained only Aβ_40_ in buffer, while another sample contained also 50 mM SDS detergent, in order to mimic a membrane environment^93^. To both samples, AgNO_3_ was added in steps of 16 µM, 56 µM, and 256 µM. The Chirascan Pro-Data v.4.4.1 software (Applied Photophysics Ltd., U.K.) was used to process the recorded spectra with an eight points smoothing filter (Savitzky-Golay).

### Fluorescence spectroscopy

A Jobin Yvon Horiba Fluorolog 3 fluorescence spectrophotometer (Longjumeau, France) was used to record the fluorescence emission intensity at 305 nm (excitation wavelength 276 nm) for samples of 10 µM Aβ peptide in 20 mM MES buffer, using a quartz cuvette with 4 mm path length at 20 °C. Measurements were carried out at pH 7.2 for different Aβ variants, i.e. Aβ_40_, Aβ_40_(NoHis) mutant, Aβ_40_(H6A) mutant, and Aβ(4–40). For Aβ_40_, measurements were carried out also at pH 5.3, and at pH 7.2 in the presence of 50 mM SDS detergent. AgNO_3_ was titrated to the samples using stock solutions of 1 mM, 2 mM, and 10 mM. The measured tyrosine fluorescence intensities were plotted against the concentration of Ag^+^ ions, and dissociation constants were evaluated by fitting the data curves to Eq. (1), i.e. the Morrison Equation ^94^:

1 $${\text{I}}={{\text{I}}}_{0}+\frac{{{\text{I}}}_{\infty }-{{\text{I}}}_{0}}{2\cdot \left[\mathrm{A\beta }\right]}\cdot \left(\left({{\text{K}}}_{{\text{D}}}+\left[{\text{Ag}}\right]+\left[\mathrm{A\beta }\right]\right)-\sqrt{{\left({{\text{K}}}_{{\text{D}}}+\left[{\text{Ag}}\right]+\left[\mathrm{A\beta }\right]\right)}^{2}-4\cdot \left[{\text{Ag}}\right]\cdot \left[\mathrm{A\beta }\right]}\right)$$

Here, I_0_ is the initial fluorescence intensity with no added Ag^+^ ions, I_∞_ is the steady-state intensity at the end of the titration, [Ag] is the concentration of added Ag^+^ ions, K_D_ is the dissociation constant, and [Aβ] is the concentration of Aβ peptide. The model assumes a single binding site. Adding a linear term for the fluorescence quenching effect of free Ag^+^ ions was considered unnecessary^95^. As no corrections for buffer conditions are made, to account for possible interactions between the silver ions and the buffer, the derived dissociation constant should be considered apparent (K_D_^App^).

### Blue native polyacrylamide gel electrophoresis (BN-PAGE)

Homogeneous solutions of SDS-stabilized Aβ_42_ oligomers, prepared as described in Section “Preparation of Aβ_42_ oligomers” with either 0, 10, 100, or 500 µM silver nitrate, were analyzed with BN-PAGE using the Invitrogen system (ThermoFisher Scientific, USA). For this purpose, 4–16% Bis–Tris Novex gels (ThermoFisher Scientific, USA) were loaded with 10 μL of samples containing either Aβ_42_ monomers or Aβ_42_ oligomers with increasing concentrations of silver, calibrated against the Amersham High Molecular Weight calibration kit for native electrophoresis (GE Healthcare, USA). The gel was first run at 4 °C for 90 min and then stained with the Pierce Silver Staining kit according to the manufacturer’s instructions (ThermoFisher Scientific, USA).

### Infrared spectroscopy

Fourier-transform infrared (FTIR) spectra of SDS-stabilized Aβ_42_ oligomers (prepared as described in Section “Preparation of Aβ_42_ oligomers” with 0–500 µM AgNO_3_) were recorded in transmission mode on a Tensor 37 FTIR spectrometer (Bruker Optik GmbH., Germany) equipped with a sample shutter and a liquid nitrogen-cooled MCT detector. The unit was continuously purged with dry air during the measurements. For measurements, 8–10 μL of the Aβ_42_ oligomer samples were put between two flat CaF_2_ discs separated by a 50 μm plastic spacer covered with vacuum grease at the periphery and assembled into an IR cuvette. The cuvette was mounted into the sample position of a sample shuttle inside the instrument’s chamber, while a metal grid positioned in the reference holder was used as background. The samples were allowed to equilibrate for at least 15 min after closing the chamber lid, to avoid interference from water vapor. The sample shuttle allowed sample and reference spectra to be recorded without opening the chamber. FTIR spectra were recorded at room temperature in the 1900–800 cm^–1^ range, using 6 mm aperture, 2 cm^–1^ resolution, a zero-filling factor of two, and 300 scans for both background and sample spectra. The light intensities above 2200 cm^–1^ were blocked with a germanium filter, and those below 1500 cm^–1^ with a cellulose membrane^96^. All spectra were analyzed and plotted using the OPUS 5.5 software. Second derivatives were calculated with a 17 cm^–1^ smoothing factor.

## Results

### Fluorescence measurements of binding affinity

**Figure 1:**
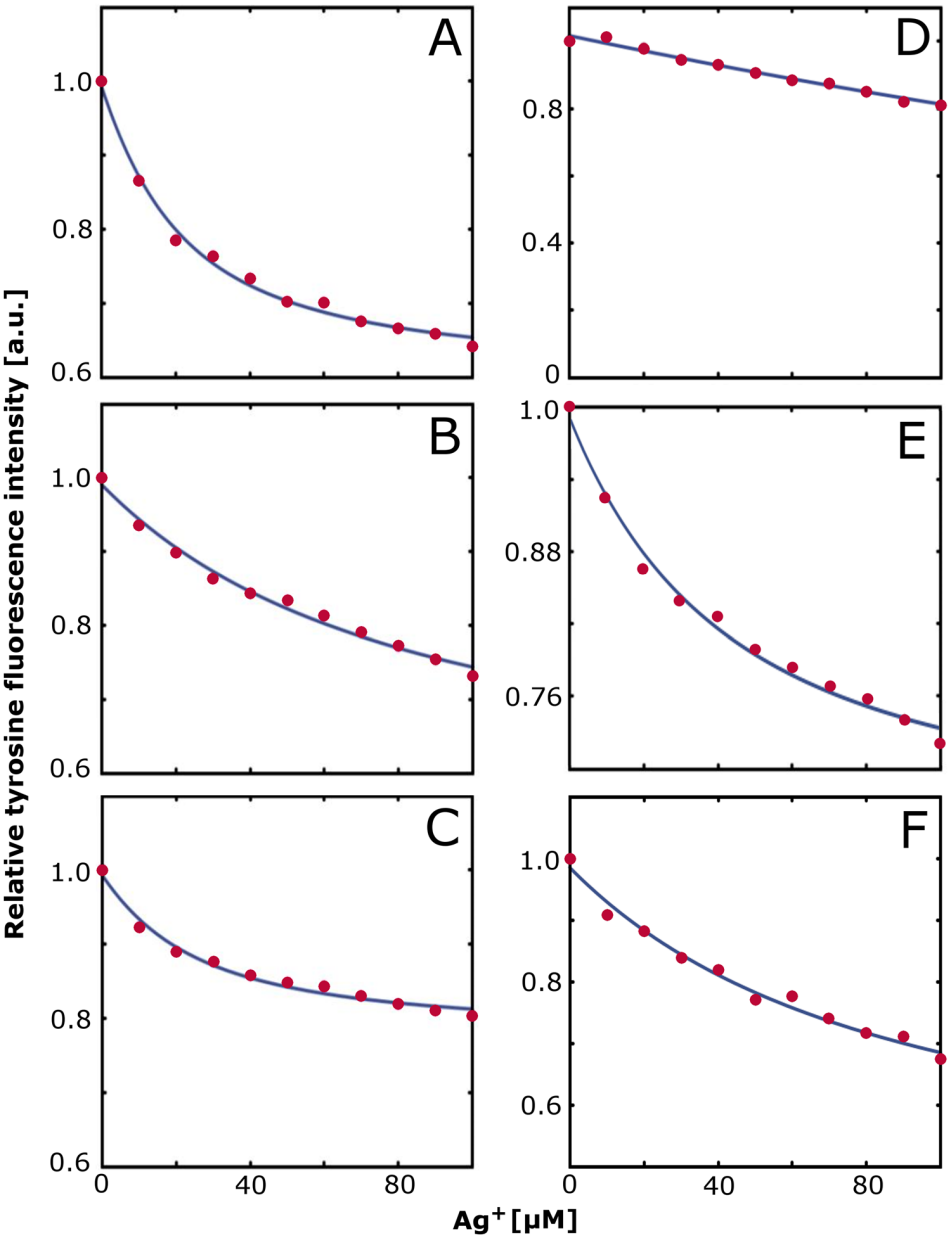
Intrinsic fluorescence of the Aβ Tyr10 residue upon titration with AgNO_3_, measured at 305 nm (excitation 276 nm) for 10 µM Aβ peptide in 20 mM MES buffer at 20 °C. (**A**) Aβ_40_ at pH 7.2; (**B**) Aβ_40_ at pH 5.3; (**C**) Aβ_40_ at pH 7.2 with 50 mM SDS; (**D**) Aβ_40_(NoHis) mutant at pH 7.2; (**E**) Aβ_40_(H6A) mutant at pH 7.2; (**F**) Aβ(4–40) at pH 7.2.

It is previously known that Ag^+^ ions can quench the intrinsic fluorescence of amino acids such as tyrosines, including the Tyr10 residue in Aβ peptides^26^. There are many molecular mechanisms that can induce such quenching^97^, and the details of Ag^+^-induced fluorescence quenching have not been fully clarified. Yet, this quenching effect can be used to derive affinity values for protein binding to Ag^+^ ions, similar to earlier studies of protein binding affinities for e.g. copper, mercury, and uranyl ions^95,98–101^. Here, measurements of the Tyr10 fluorescence upon titrations with silver ions were used to derive binding affinities for Aβ·Ag^+^ complexes, formed with different Aβ peptide variants, and measured under different conditions. Most of these measurements produced binding curves that could be fitted with Eq. (1) (Fig. 1), and thereby yield the estimated binding constants (K_D_) shown in Table 1.

**Table 1:** Apparent K_D_ values (K_D_^App^) in µM for different Aβ·Ag^+^ complexes, obtained by fitting Eq. (1) to the fluorescence intensity curves shown in Fig. 1.

Peptide type and condition	Repeat 1	Repeat 2	Repeat 3	Mean K_D_^App^ value (µM)
Aβ_40_ pH 7.2	17.8	41.8	15.9	25 ± 12
Aβ_40_ pH 7.2 + 50 mM SDS	11.9	19.5	44.6	25 ± 15
Aβ_40_ pH 5.3	76.9	232.3	78.7	129 ± 73
Aβ_40_(NoHis) pH 7.2	–	–	–	–
Aβ_40_(H6A) pH 7.2	36.7	28.5	37.0	34 ± 4
Aβ(4–40) pH 7.2	81.0	113.5	10.4	68 ± 44

For the wt Aβ_40_ peptide, the average K_D_ value is 25 ± 12 µM at pH 7.2 (Fig. 1A). This is similar to previously reported K_D_ values of 4–16 µM^26^. At pH 5.3 the K_D_ value is 129 ± 73 µM (Fig. 1B). Because the main difference between neutral and acidic pH is that His residues become protonated at low pH, the weaker binding affinity at pH 5.3 suggests that His residues are involved in the metal coordination. This is supported by the results for the Aβ_40_(NoHis) mutant at pH 7.2, where only a weak linear quenching effect is observed (Fig. 1D), corresponding to concentration-dependent quenching only from free (non-bound) silver ions^95^. It was therefore not possible to fit these data to Eq. 1, and no binding constant was derived for the Aβ_40_(NoHis) mutant (Table 1). For the Aβ_40_(H6A) mutant and the truncated Aβ(4–40) variant, titrations with Ag^+^ ions at pH 7.2 produced proper binding curves (Fig. 1E,F), yielding average K_D_ values of 34 ± 4 µM and 68 ± 44 µM, respectively (Table 1). Because these values are not statistically different from the 25 ± 12 µM obtained for the wt Aβ_40_ peptide (Table 1), it appears that Ag^+^ binding is not much affected when only one histidine (His6) is removed, or when the first three residues (including anionic Asp1 and Glu3) are removed. Measurements on wt Aβ_40_ peptide at pH 7.2 in the presence of SDS micelles yielded a K_D_ value of 25 ± 15 µM (Fig. 1C). As the latter value is almost identical to the K_D_ value for wt Aβ_40_ peptide without SDS micelles (Table 1), these results confirm earlier reports that membrane-mimicking environments do not have a strong effect on Aβ metal-binding^98,102^.

### NMR spectroscopy

**Figure 2:**
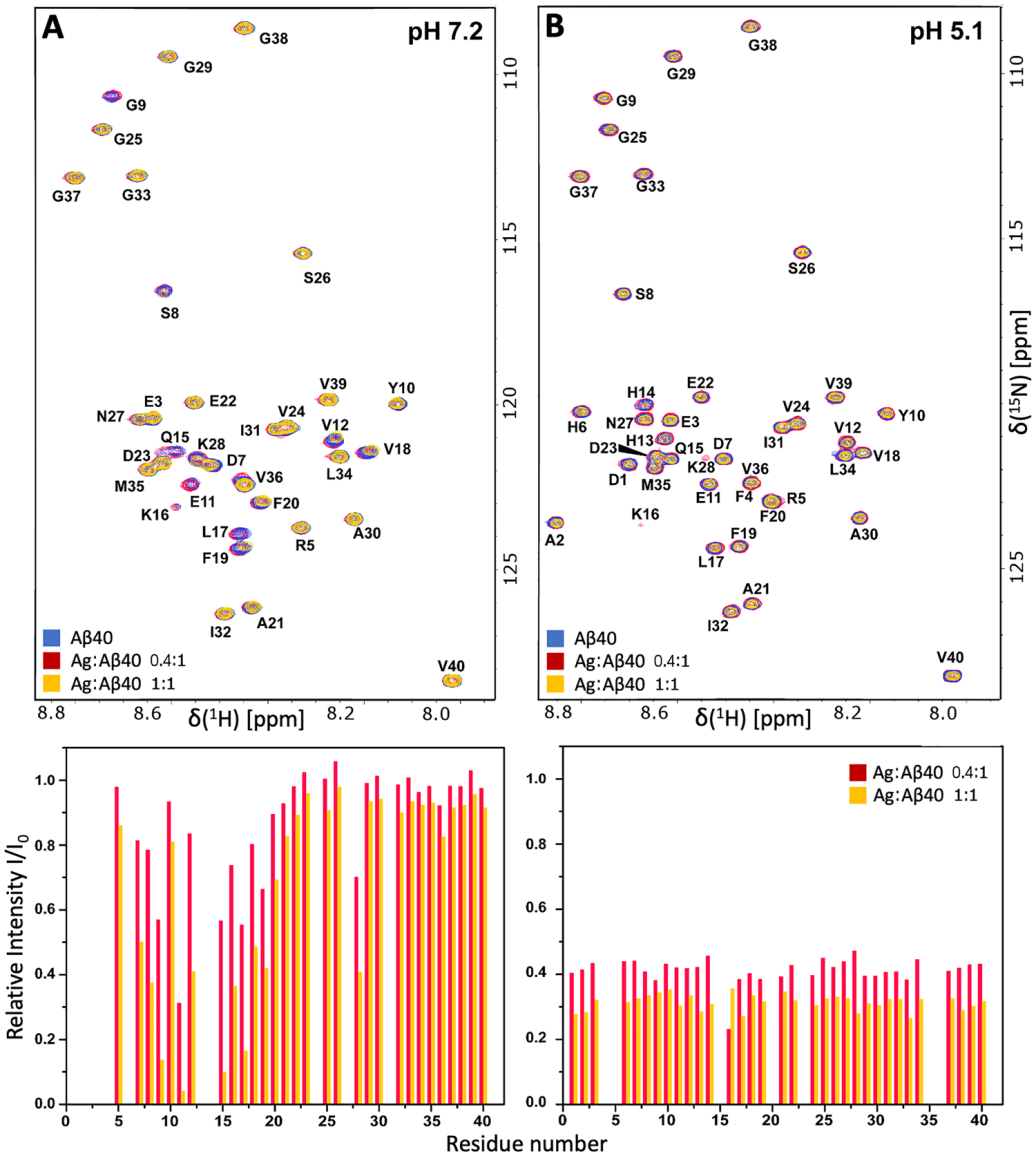
2D ^1^H,^15^N-HSQC NMR spectra recorded at 5 °C for 92.4 µM monomeric ^15^N-labeled Aβ_40_ peptide in 20 mM MES buffer (90/10 H_2_O/D_2_O), either at pH 7.2 (**A**) or at pH 5.1 (**B**). Blue: Aβ_40_ in buffer only. Red: + 37 µM AgNO_3_ (0.4:1 Ag^+^:Aβ ratio) Yellow: + 92.4 µM AgNO_3_ (1:1 Ag^+^:Aβ ratio). The bar diagrams show relative crosspeak intensities after additions of 37 µM AgNO_3_ (red) and 92.4 µM AgNO_3_ (yellow).

High-resolution NMR spectroscopy was conducted to monitor residue-specific interactions between Ag^+^ ions and monomeric Aβ_40_ peptides. Figure 2 shows 2D ^1^H,^15^N-HSQC spectra for the amide cross-peak region for 92.4 µM ^15^N-labeled Aβ_40_ peptides, at either pH 7.2 or pH 5.1, measured before and after addition of silver nitrate.

At pH 7.3, addition of first 37 µM and then 92.5 µM silver nitrate (0.4:1 and 1:1 Ag^+^:Aβ_40_ ratio, respectively) induces a concentration-dependent loss of amide cross-peak intensity that is most pronounced around residues 10–15 (Fig. 2A). This shows that there is a specific binding site for Ag^+^ ions in the N-terminal segment. Because Ag^+^ ions are diamagnetic, the reduction in crosspeak intensity is likely caused by intermediate chemical exchange, on the NMR time-scale^103^, between the Aβ_40_·Ag^+^complex and free Aβ_40_ peptides, similar to the effects observed when Aβ peptides interact with diamagnetic Zn^2+^ ions^26,104,105^. For interactions with e.g. Cu^2+^ and Ni^2+^ ions, there is also a paramagnetic quenching effect on the NMR signals^92,102^. As the relative crosspeak intensities for the C-terminal residues remain at around 1.0 after addition of silver nitrate (Fig. 2A), it appears that there is little interaction with Ag^+^ ions in this region.

At pH 5.1, addition of 37 µM silver nitrate uniformly reduces the amide crosspeak intensities to around 0.4, while addition of 92.5 µM silver nitrate uniformly reduces the crosspeak intensities to around 0.3 (Fig. 2B). This uniform and concentration-dependent intensity loss shows that there is no residue-specific binding of Ag^+^ ions to the Aβ_40_ peptides at acidic pH. When comparing the NMR spectra of Aβ_40_ peptides alone at neutral (Fig. 2A) and acidic (Fig. 2B) pH values, i.e. without added silver nitrate, NMR crosspeaks for additional residues (i.e., D1, A2, H6, H13, and H14) can be observed at pH 5.1 (Fig. 2B), likely due to slower proton exchange at acidic pH^92^.

### Circular dichroism (CD) spectroscopy on Aβ secondary structure

**Figure 3:**
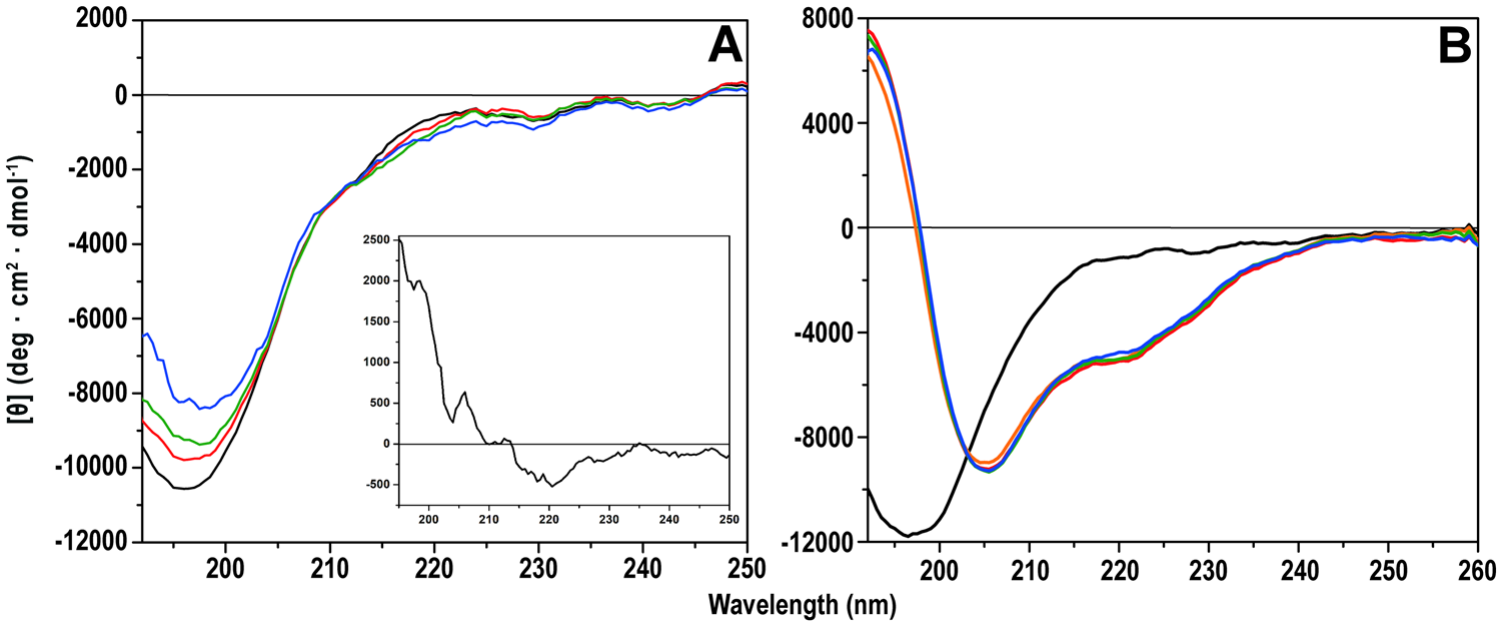
CD spectra of 10 μM Aβ_40_ peptide in 20 mM phosphate buffer, pH 7.3. AgNO_3_ was titrated to the peptide, either in (**A**): buffer only, or (**B**): with 50 mM SDS present. Black: Aβ_40_ in buffer. Orange: + 50 mM SDS (in B only). Red: + 16 µM AgNO_3_. Green: + 56 µM AgNO_3_. Blue: + 256 µM AgNO_3_. The insert in (**A**) shows a difference spectrum, obtained by subtracting the spectrum with 256 µM AgNO_3_ (blue) from the spectrum with only Aβ_40_ in buffer (black).

CD spectroscopy was used to investigate possible effects of silver ions on the secondary structure of Aβ_40_ peptides, both in aqueous solution and in the presence of SDS micelles mimicking a membrane environment^93^. The CD spectrum for Aβ_40_ monomers in aqueous buffer shows a minimum around 196–198 nm (Fig. 3A), typical for a random coil ensemble with a 3_1_-helix (polyproline II) component^106^. Titration with silver ions produces a concentration-dependent two-state structural transition around an isodichroic point at approximately 212 nm (Fig. 3A). The difference spectrum, created by subtracting the last spectrum in the titration from the first, shows a minimum around 220 nm (Fig. 3A, insert). This suggests that the observed transition is from a mixed random coil ensemble to a random coil ensemble with slightly increased β-sheet structure, and slightly reduced 3_1_-helix component^106^.

In the presence of SDS micelles, the Aβ_40_ CD spectrum has minima at 208 and 222 nm, typical for α-helical secondary structure (Fig. 3B). This confirms previous studies showing that the central and C-terminal Aβ regions adopt α-helical conformations in membrane-like environments^19,93,98,107^. In this membrane-like environment (SDS micelles), addition of Ag^+^ ions does not induce any significant changes in the Aβ_40_ secondary structure (Fig. 3B).

### AFM imaging

**Figure 4:**
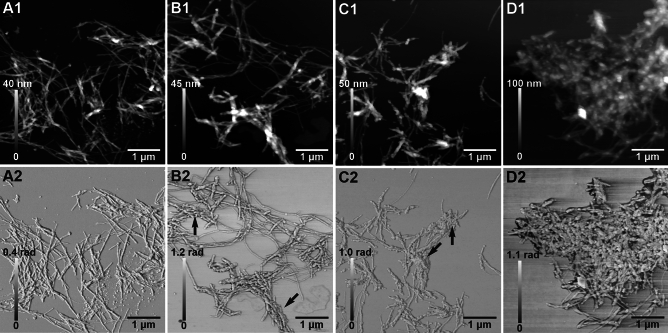
AFM images for 10 µM Aβ_40_ peptides together with different concentrations of AgNO_3_, incubated with agitation for 72 h at 37 °C in 5 mM MES buffer, pH 7.3. (**A**) 0 µM AgNO_3_; (**B**) 2.5 µM AgNO_3_; (**C**) 5 µM AgNO_3_; (**D**) 30 µM AgNO_3_. Top row: height profiles. Bottom row: mechanical phase images.

To study the effects of Ag^+^ ions on Aβ_40_ aggregation and fibril morphology, AFM images were recorded for Aβ_40_ aggregates formed after 72 h incubation in the presence and the absence of silver nitrate (Fig. 4). Without Ag^+^ ions, 10 µM Aβ_40_ peptide formed typical amyloid fibrils that are less than 10 nm thick and tangled together (Fig. 4A). This is in line with previously published studies on Aβ fibrils formed in vitro^6,31,102,108^. Similar fibrils were formed in the presence of sub-stoichiometric amounts of silver nitrate, i.e. 2.5 µM and 5 µM (Fig. 4B,C). In the presence of 30 µM AgNO_3_, on the other hand, some changes are observed. The individual fibrils appear to have the same shape and size as those formed without silver ions, but the fibrils are now much more tangled together (Fig. 4D). This suggests that the silver ions do not affect how the fibrils are formed, but rather how they are organized after formation. The 30 µM Ag^+^ concentration is of the same order of magnitude as the K_D_ value for the Ag^+^·Aβ_40_ complex, i.e. 25 ± 12 µM (Table 1). Thus, at this concentration about half the Aβ_40_ peptides are in complex with Ag^+^ ions, which should be enough to induce any structural effect that might exist.

### BN-PAGE and FTIR analysis of Aβ_42_ oligomers

**Figure 5:**
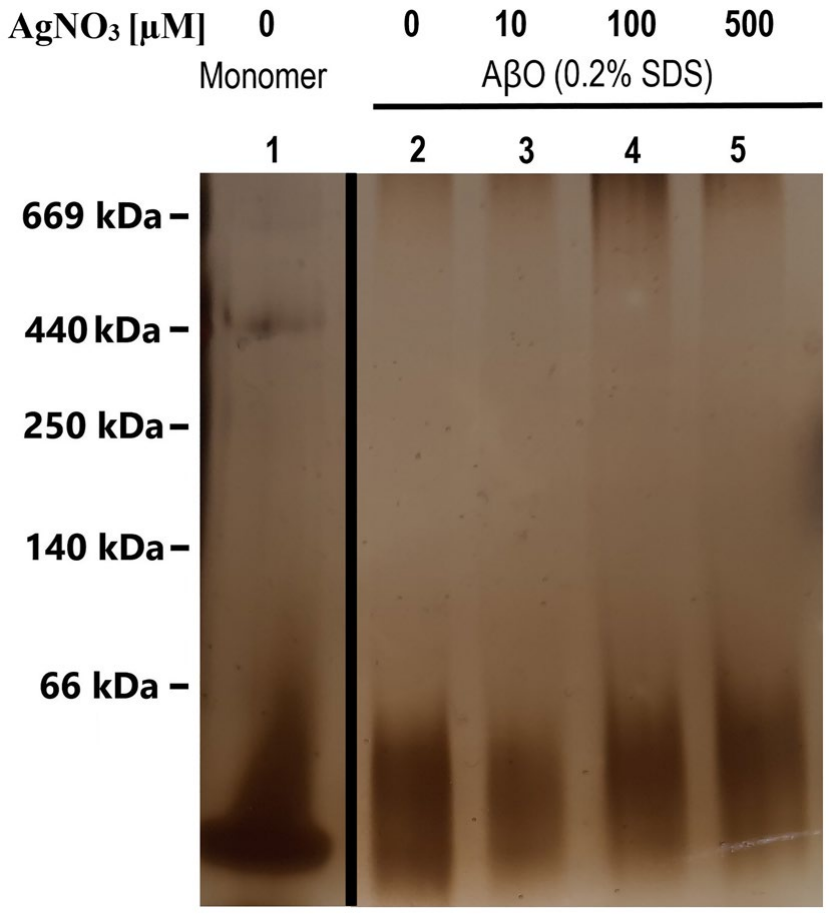
BN-PAGE gel showing the effects of different concentrations of silver nitrate on the formation of SDS-stabilized Aβ_42_ oligomers (formed with 100–120 µM monomeric Aβ_42_ peptide). Lane 1: Aβ_42_ monomers. Lanes 2–5 Aβ_42_ oligomers prepared with 0, 10, 100, and 500 µM AgNO_3_, respectively.

Aβ_42_ forms stable oligomers in the presence of sub-micellar concentrations of SDS^83^. We here used BN-PAGE analysis and FTIR spectroscopy to investigate possible effects of silver ions on the structure of such oligomers. Because they are stabilized by SDS only and not by cross-linking, BN-PAGE was chosen for analysis instead of SDS-PAGE, as the high (> 1%) SDS concentrations used in SDS-PAGE sample buffers might disrupt SDS-stabilized oligomers^109,110^. Thus, oligomers with an average MW around 16–20 kDa (Fig. 5, Lane 2) were prepared from Aβ_42_ monomers together with 0.2% (6.9 mM) SDS detergent and with either 0, 10, 100, or 500 µM AgNO_3_ as described in Section “Preparation of Aβ_42_ oligomers”.

The BN-PAGE analysis shows that the average sizes, amounts, and size distributions are approximately the same for Aβ_42_ oligomers formed with and without silver nitrate (Fig. 5, Lanes 2–5; Supp. Fig. S1). In particular, the peptide band for oligomers prepared in the presence of 500 µM AgNO_3_ is very similar to the band for oligomers formed without silver ions.

Corresponding results were obtained with the FTIR measurements. The IR amide I region (1700–1600 cm^-1^) is very sensitive to changes in the protein/peptide backbone conformation, corresponding to differences in secondary structure^111–113^. For Aβ_42_ oligomers, the spectral position and the width of the main β-sheet band have been shown to correlate with oligomer size and structural heterogeneity, respectively^85^.

**Figure 6:**
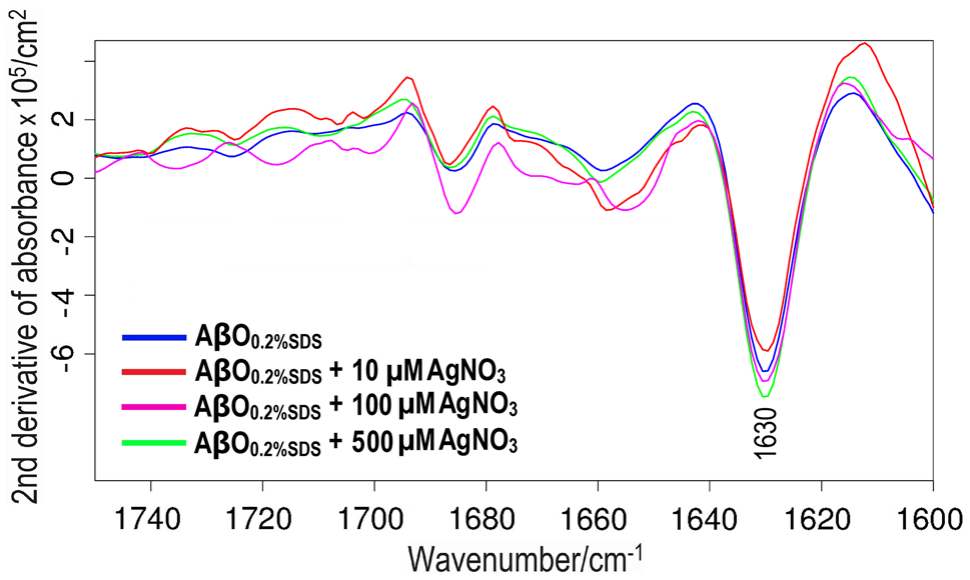
Second derivatives of infrared absorbance spectra for SDS-stabilized Aβ_42_ oligomers alone (blue) and Aβ_42_ oligomers formed together with either 10 µM (red), 100 µM (purple), or 500 µM (green) silver nitrate.

The Aβ_42_ oligomers prepared in absence of AgNO_3_ display two major bands in the amide I region: a high-intensity, low-wavenumber band near 1630 cm^-1^ and a smaller, high-wavenumber band near 1686 cm^-1^^85^. These two bands are typical for anti-parallel β-sheet conformation^21,114,115^. We have earlier shown a relationship between the position and width of the β-sheet main band, and the size and homogeneity of Aβ_42_ oligomers^85^. For Aβ_42_ oligomers prepared together with different amounts of AgNO_3_, the positions for the main band shown in Fig. 6 are: 1630.1 cm^-1^ (0 µM Ag^+^); 1629.9 cm^-1^ (10 µM Ag^+^); 1630.1 cm^-1^ (100 µM Ag^+^); 1630.1 cm^-1^ (500 µM Ag^+^). Thus, no systematic changes in the main band position are observed, not even for the highest amount of 500 µM Ag^+^ (Fig. 6). These observations agree with the BN-PAGE results (Fig. 5), and show that silver ions have no significant effect on the following properties of Aβ_42_ oligomers: size, secondary structure, β-sheet planarity, and number of strands in the β-sheets.

## Discussion

The molecular interactions underlying the antimicrobial properties of silver ions and silver nanoparticles are still unclear, and a possible role of silver in neurodegenerative diseases remains to be clarified^48,62,79,81^. Because Ag^+^ ions display similar binding properties to proteins as Cu^+^ ions, they have often been applied as probes for studying molecular interactions between proteins and Ag^+^/Cu^+^ ions^56–58^. Thus, Ag^+^ ions have been shown to bind to amyloidogenic proteins such as the prion protein^116^ and insulin^117^. Furthermore, amyloid-carbon membranes engineered to purify water are reported to bind heavy metal ions including Ag^+^^118^. For Alzheimer’s disease, silver nanoplates have been shown to dissolve aggregated Aβ fibrils^119^, and several studies have reported on Aβ peptide interactions with metal ions^101,102,120–124^ including silver ions^26,59,60^. We here interpret the current results on Aβ-silver interactions in the light of these earlier findings.

### Residue-specific binding of Ag^+^ ions to different Aβ peptide variants

The fluorescence measurements show that at neutral pH, Ag^+^ ions bind to wt Aβ_40_ peptide with an affinity of 25 ± 12 µM (Fig. 1A; Table 1). This value is similar to the previously reported affinity values in the 4–16 µM range measured with both NMR and fluorescence spectroscopy^26^. Thus, Ag^+^ ions appear to bind Aβ peptides with roughly similar affinity as Zn^2+^ and Ni^2+^ ions, i.e. around 1–50 µM^102,104,125,126^, but weaker than Cu^2+^ ions which have sub-micromolar binding affinities^95,99,125^. In order for affinity values to be properly compared, they should have been obtained with the same method, as both the sample conditions and measurement technique can influence the outcome^99^. When fluorescence measurements of binding affinities for the Aβ·Cu^2+^ complex are corrected for buffer effects, values around 1 nM are obtained^99,125^.

Our NMR results demonstrate that Ag^+^ ions at neutral pH bind to the N-terminal segment of the Aβ_40_ peptide (Fig. 2A), confirming earlier results^26^. At acidic pH, the specific binding to the N-terminal segment is lost (Fig. 2B), and the binding affinity is weaker, i.e. 129 ± 73 µM (Fig. 1B). Because histidines become protonated at low pH^127,128^, and thereby less suitable for binding cations^92,95^, these results support previous studies suggesting that Ag^+^ ions are coordinated by Aβ His residues^26^. This is supported also by the fluorescence measurements for the Aβ_40_(NoHis) mutant, where no binding to Ag^+^ ions was observed (Fig. 1D). This is very reasonable given that Ag^+^ ions generally prefer donor atoms and binding residues in the order of S > N > O and Cys > Met > His > Lys > Val, respectively^50^, in addition to electrostatic interactions provided by anionic Asp and Glu residues^52,129^. The Aβ_40_ peptide has no cysteines, and only one methionine—Met35—with no other suitable metal-binding residues around it. Coordination of Ag^+^ ions with at least some of His6, His13, and His14 is therefore logical.

Earlier investigations have shown that divalent metal ions such as Cu^2+^ and Zn^2+^ usually do not bind to Aβ peptides with a single binding conformation^120,130^. Instead, different alternating binding configurations appear to exist, with Asp1, Glu3, His6, Asp7, Glu11, His13, and His14 as potential binding residues, typically in tetrahedral conformation^104,120,130,131^. For example, one reported structure shows the Aβ(1–16) fragment coordinating Zn^2+^ ions via the His6, His13, and His14 aromatic side chains together with the carboxylate of the Glu11 side chain^132^. A later study found such coordination to be the starting point for Zn^2+^-induced Aβ dimerization, where one Zn^2+^ ion then became coordinated by two His14 and two Glu11 residues, i.e. from two different Aβ peptides^133^.

In contrast to Zn^2+^ ions, Ag^+^ ions generally prefer linear coordination^134^, although trigonal or tetragonal binding modes are possible. Ag^+^ ions furthermore display similar binding properties to proteins as monovalent Cu+ ions^56–58^, and previous studies have suggested that Cu^+^ ions bind to the Aβ N-terminal segment via two histidines, in different possible combinations of the three available His residues^135–137^. It appears reasonable that Ag^+^ ions coordinate to Aβ peptides in a similar way, i.e. with a bis-his binding configuration. This would explain why the K_D_ value for Ag^+^ binding to the Aβ_40_(H6A) mutant (34 ± 4 µM; Fig. 1E; Table 1) is not significantly different from the value for wt Aβ_40_ (25 ± 12 µM; Table 1). That is, if Ag^+^ ions favour coordination via two histidines, removing one His residue (His6) would have little effect on Ag^+^ binding as long as the other two histidines (His13 and His14) are present.

Binding of Ag^+^ to Aβ_40_ peptides in the presence of SDS micelles produces a K_D_ value of 25 ± 15 µM, i.e. very similar to the 25 ± 12 µM reported for wt Aβ_40_ peptides in aqueous solution (Fig. 1; Table 1). This is in line with earlier studies showing little or no effect of SDS micelles on Aβ binding affinity for Ag^+^, Cu^2+^ and Ni^2+^ ions^98,102^. While the more hydrophobic central and C-terminal Aβ segments insert themselves into SDS micelles^19^, the hydrophilic N-terminal segment remains positioned outside the micelles where it is free to bind e.g. metal ions^98^. Because metal ions typically bind to the N-terminal segment, it is generally agreed that Aβ variants that differ only in the C-terminal segment, such as Aβ_40_ and Aβ_42_, have virtually identical metal-binding properties.

For the truncated Aβ(4–40) variant the binding affinity is 68 ± 44 µM (Table 1). Due to the large error, this value is not statistically different from the 25 ± 12 µM observed for wt Aβ_40_ (Table 1). By removing the first three amino acids, His6 in the full-length Aβ_40_ sequence becomes residue number three in the Aβ(4–40) sequence, thereby creating a so-called ATCUN (Amino Terminal Cu and Ni) binding motif. This motif has very strong binding affinity towards metal ions such as Cu^2+^ and Ni^2+^^138–140^, but has been reported to display weaker binding to Cu^+^ and Ag^+^ ions^56–58^. The current results support the idea that ATCUN motifs do not provide advantageous binding to Ag^+^ ions, probably due to Ag^+^ ions (and also Cu^+^ ions) coordinating to Aβ peptides with a different geometry than Cu^2+^ and Ni^2+^ ions (as discussed above). The results obtained for the Aβ(4–40) peptide furthermore suggest that the anionic Asp1 and Glu3 residues, which are deleted in the truncated variant, do not contribute strongly to Ag^+^ binding (Table 1).

### Effects of silver ions on Aβ structure and aggregation

Previous studies have shown that binding of Ag^+^ ions to the Aβ_40_ peptide can affect its secondary structure^26,59^. Here, our CD measurements show that in aqueous buffer, binding of Ag^+^ ions to Aβ_40_ slightly promotes β-sheet conformation (Fig. 3A). Similar structural conversions have been observed with CD spectroscopy for Aβ binding to e.g. Cu^2+^, Zn^2+^, and Ni^2+^ ions^92,102,141^. The increased β-sheet content is consistent with earlier reports that the Aβ·Ag^+^ complex is more compact than free Aβ peptides, with a structure that is unfavorable for fibril formation^26^.

For Aβ_40_ peptides positioned in SDS micelles, which are a simple membrane-mimicking system^93,107^, binding of Ag^+^ ions does not induce any structural alterations (Fig. 3B). Ag^+^ ions furthermore have no apparent effects on the secondary structure or size distributions of Aβ_42_ oligomers (Figs. 5, 6). A similar lack of structural effects has previously been reported by Li^+^ ions, another monovalent species^124^. In contrast, structural effects on Aβ_42_ oligomers and on SDS-bound Aβ_40_ monomers have been observed for divalent Cu^2+^ and Ni^2+^ ions^98,102^. It is possible that the weaker electrostatic interactions elicited by monovalent ions are not able to induce the same structural changes as those caused by divalent ions. But it is also possible that Ag^+^ ions have different binding configurations to Aβ peptides than e.g. Cu^2+^ and Ni^2+^ ions, and therefore create different structural effects.

It is generally accepted that the biological properties of a protein depend on its structure. Because Aβ oligomers are considered to be the main toxic species in AD^11,12^, where cell membrane disruption could be one of the toxic mechanisms^142^, the observation that Ag^+^ ions appear to have no structural effects on Aβ_42_ oligomers allows us to suggest that Ag^+^ ions should have limited effect on Aβ toxicity.

This suggestion is supported by Ag^+^ ions having almost no effect on Aβ fibril structure (Fig. 4), but only on the organization of fibrils after they have formed (Fig. 4), an observation in line with earlier studies^26^. In contrast, multivalent metal ions such as Cu^2+^, Zn^2+^, Mn^2+^, Ni^2+^, Hg^2+^, Pb^4+^ and UO_2_^2+^ typically re-direct the aggregation process towards formation of non-amyloid aggregates that are amorphous and unstructured^3,101,102,122,123,126^. In this respect Ag^+^ ions resemble Li^+^ ions, which also are monovalent and have weak effects on Aβ structure and aggregation^124^.

## Conclusions

Silver ions bind wt Aβ_40_ peptides with a binding affinity of 25 µM in pH 7.2 MES buffer at 20 °C, both in the absence and in the presence of SDS micelles. The N-terminal His residues are involved as coordinating groups, likely in bis-his configuration. Weaker Ag^+^ binding is observed for the wt Aβ_40_ peptide at acidic pH and for an Aβ_40_ mutant without histidines. No significant change in binding affinity is observed for the Aβ_40_(H6A) mutant or for the truncated Aβ(4–40) variant that has an ATCUN binding motif. In contrast to divalent metal ions such as Ni^2+^, monovalent Ag^+^ ions do not induce any changes in the size or structure of Aβ_42_ oligomers, which are considered to be the main toxic species in Alzheimer’s disease. We therefore suggest that Ag^+^ ions have a minor influence on Aβ toxicity.

## Supplementary Information

Supplementary Information 1.

## Data Availability

All data generated and analysed during this study are included in this published article and in the supplementary information file.
