# Supplementary material for: Molecular interactions, structural effects, and binding affinities between silver ions (Ag+) and amyloid beta (Aβ) peptides
Source: Sci Rep. 2025 Feb 13;15:5439. doi: 10.1038/s41598-024-59826-6 (PMC11825922; doi:10.1038/s41598-024-59826-6)
Supplement: Supplementary file 1 — Supplementary Information 1. [file 41598_2024_59826_MOESM1_ESM.pdf]

Supporting Information

Molecular interactions, structural effects, and binding affinities between silver ions (Ag^+) and amyloid beta (Aβ) peptides

Amanda L. Lakela[1,†], Elina Berntsson[1,2,3,†], Faraz Vosough[1], Jüri Jarvet[1,2,4], Suman Paul[1], Andreas Barth[1], Astrid Gräslund[1,2,*], Per M. Roos[5,6], Sebastian K.T.S. Wärmländer[2,7,*]

[1] Department of Biochemistry and Biophysics, Arrhenius Laboratories, Stockholm University, 10691 Stockholm, Sweden.
[2] CellPept Sweden AB, Kvarngatan 10B, 11847 Stockholm, Sweden
[3] Dept. of Chemistry and Biotechnology, Tallinn University of Technology, 19086 Tallinn, Estonia.
[4] The National Institute of Chemical Physics and Biophysics, Tallinn, Estonia.
[5] Institute of Environmental Medicine, Karolinska Institutet, 17177 Stockholm, Sweden.
[6] University Healthcare Unit of Capio St. Göran Hospital, 11281 Stockholm, Sweden.
[7] Chemistry Section, Arrhenius Laboratories, Stockholm University, 10691 Stockholm, Sweden.

[†] These two authors contributed equally and should both be considered first authors.
***** Corresponding authors. E.-mail: astrid@dbb.su.se; seb@student.su.se

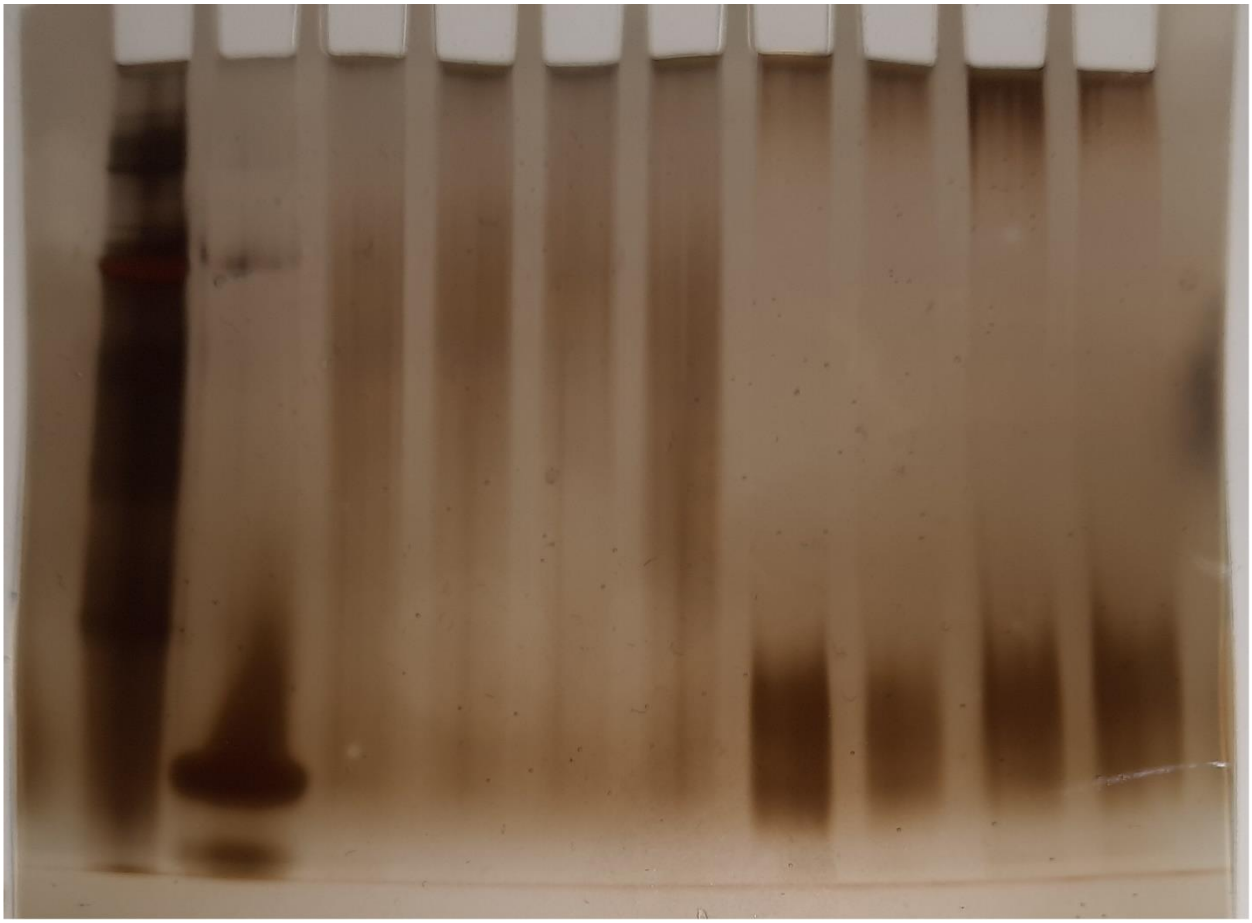

Supp. Fig. S1. Original image of the BN-PAGE gel shown in Fig. 5 in the main manuscript. Lane 1. Ladder with high molecular weight reference proteins from GE Healthcare, USA; Lane 2. $A\beta_{42}$ monomers; Lanes 3-6. $A\beta_{42}$ oligomers prepared with 0.05 % SDS and 0, 10, 100, and 500 µM $AgNO_3$, respectively.; Lanes 7-10. $A\beta_{42}$ oligomers prepared with 0.2 % SDS and 0, 10, 100, and 500 µM $AgNO_3$, respectively. Image by F.V.
